# Interaction of SNARE Mimetic Peptides with Lipid bilayers: Effects of Secondary Structure, Bilayer Composition and Lipid Anchoring

**DOI:** 10.1038/s41598-019-43418-w

**Published:** 2019-05-22

**Authors:** Swapnil Wagle, Vasil N. Georgiev, Tom Robinson, Rumiana Dimova, Reinhard Lipowsky, Andrea Grafmüller

**Affiliations:** Department of Theory and Bio-Systems, Max Planck Institute of Colloids and Interfaces, Science Park Golm, 14424 Potsdam, Germany

**Keywords:** Computational biophysics, Membrane biophysics

## Abstract

The coiled-coil forming peptides ‘*K*’ enriched in lysine and ‘*E*’ enriched in glutamic acid have been used as a minimal SNARE mimetic system for membrane fusion. Here we describe atomistic molecular dynamics simulations to characterize the interactions of these peptides with lipid bilayers for two different compositions. For neutral phosphatidylcholine (PC)/phosphatidylethanolamine (PE) bilayers the peptides experience a strong repulsive barrier against adsorption, also observed in potential of mean force (PMF) profiles calculated with umbrella sampling. For *peptide K*, a minimum of −12 *k*_*B*_*T* in the PMF provides an upper bound for the binding free energy whereas no stable membrane bound state could be observed for *peptide E*. In contrast, the electrostatic interactions with negatively charged phosphatidylglycerol (PG) lipids lead to fast adsorption of both peptides at the head-water interface. Experimental data using fluorescently labeled peptides confirm the stronger binding to PG containing bilayers. Lipid anchors have little effect on the peptide-bilayer interactions or peptide structure, when the peptide also binds to the bilayer in the absence of a lipid anchor. For *peptide E*, which does not bind to the PC bilayer without a lipid anchor, the presence of such an anchor strengthens the electrostatic interactions between the charged side chains and the zwitterionic head-groups and leads to a stabilization of the peptide’s helical fold by the membrane.

## Introduction

**Figure 1:**
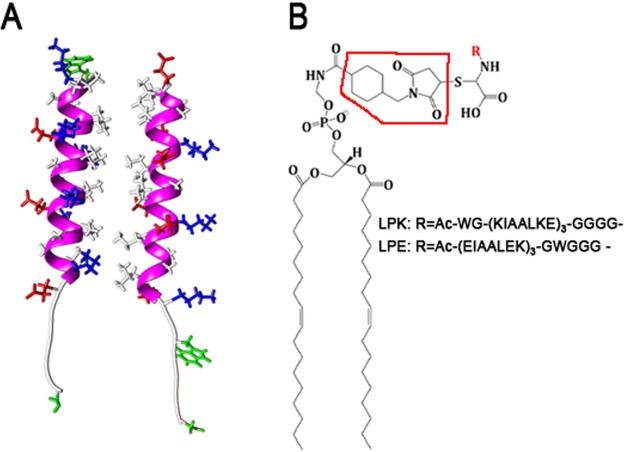
(**A**) Coiled-coil structure of *peptides K* and *E* with the helical backbone in magenta and the extended linker sequence shown in light grey. Side chains are drawn in red = negative, blue = positive, white = hydrophobic residues, TRP and CYS side chains are highlighted in green; and (**B**) Structure of the maleimide-containing lipid anchor for *LPE* and *LPK*. The maleimide group is indicated by the red frame.

Membrane fusion is a key process in cellular biology. In eukaryotes, most intracellular membrane fusion events are orchestrated by SNARE (soluble N-ethylmaleimide-sensitive factor attachment protein receptor) complexes. The proposed working mechanism of these protein complexes is the zipper-like assembly of a coiled-coil bundle of four alpha helices, which assists membrane docking and overcomes the water barrier between the two membranes^1,2^. The SNARE protein machinery has inspired the design of simplified model fusion systems based on lipid vesicles, which aim to mimic the zipper like folding mechanism using DNA^3–7^, PNA^8–10^, peptides^11–15^ or other molecules^16^. The complementary coiled-coil forming peptides shown in Fig. 1 are referred to as ‘*K*’ (KIAALKE)_3_ and ‘*E*’ (EIAALEK)_3_ according to the abundance of lysine (K) and glutamic acid (E) residues found in their sequences. *Peptides E* and *K* have been investigated as a successful fusogenic system^13–15,17^. They form heterodimer coiled-coils, with a hydrophobic core surrounded by pairs of oppositely charged residues on either side. They are incorporated into lipid vesicles by attachment to a lipid anchor, either via a polyethylene glycol (PEG) spacer^14^ or using maleimide chemistry^17,18^. In some experiments, extra residues are added to the peptides in order to make them more flexible or to label them for fluorescence and/or FRET studies. Although, the *peptides E* and *K* have been shown to produce vesicle fusion, the fraction of successful fusion events is typically low compared to fusion induced by SNARE complexes. A molecular level understanding of the interactions of these peptides is therefore highly desirable, both for the development of more efficient artificial fusion systems and as a step towards a better understanding of the SNARE mediated fusion process.

A number of studies have investigated different variations of this system, studying the effect of peptide length^19^, orientation^20^, linker and anchor types^21–23^ and attachment point^24^ on the peptides’ helicity, coiled-coil forming ability and efficiency at generating vesicle fusion. The fusion mechanism, which has been suggested based on these studies, proposes slightly different roles for the two peptides, in which *peptide K* interacts with the membrane in addition to forming the coiled-coil with *peptide E* and thus plays a role in destabilizing the membrane for fusion^25^. This observation agrees also with the classification of alpha-helical amphipathic/amphiphilic peptides, and their binding patterns^26–29^, by which *peptide K* belongs to the so-called category A1, which is expected to interact with zwitterionic bilayers, whereas the charge distribution on *peptide E* is not favorable for binding to the latter type of bilayers.

A detailed picture of the molecular interactions can be gained with the help of molecular simulations. In many cases, coarse-grained (CG) models have proven quite successful to model lipid bilayer systems, and have been extensively applied to understand the role of the lipid bilayer in the fusion process^30,31^. A reliable representation of proteins at the coarse grained scale on the other hand is ‘tricky’ and typically requires the secondary structure to be restrained, as for instance in the widely used MARTINI force field^32–34^. As a consequence, there are much fewer studies modeling the role of proteins in membrane fusion.

Nevertheless, several simulation studies also characterize the interactions of the fusion *peptides E* and *K* with the membrane. A first CG study using the MARTINI force field has reported that both peptides insert into the lipid bilayer^35^, and speculated about the implications of this for the fusion mechanism. However, the very strong interactions are at odds with experimental results finding almost no interactions of *peptide E* and only weak interactions of *peptide K* with the bilayer^25,36,37^. A more recent study has shown that the use of polar water with the MARTINI force field strongly reduces the tendency of the peptides to adsorb^38^. In those simulations, only *peptide K* is found to embed in the membrane. Finally, all atom molecular dynamics (MD) simulations have been used to assess the membrane interactions of the adsorbed peptide^25,38^ and to determine the affinity of the fusion peptides to the bilayer with umbrella sampling^39^. These simulations suggest that both peptides adsorb equally strongly at the head-group water interface, but do not embed in the bilayer. In addition the adsorbed peptides are partially unfolded, which is also observed in other atomistic simulations^35^, whereas the peptides are restrained to remain helical in the CG simulations.

To obtain a clearer picture of the factors affecting peptide-bilayer interactions and the behavior of the peptides close to and anchored to the membrane, we have performed a series of all atom MD simulations and free energy calculations of the peptides. Our study involves modeling the peptides both in contact with two lipid bilayers of different compositions, and in solution. The bilayer compositions used here include a neutral phosphatidylcholine (PC)/Phosphatidylethanolamine (PE) bilayer, which corresponds to the composition of the model membranes commonly used for the majority of both experimental and theoretical studies of the system. The second lipid composition is representative of the liquid disordered phase in phase-separated vesicles composed of a ternary lipid mixture with charged lipids^40^ and used in an assay for phase specific fusion^41^. This bilayer contains negatively charged glycero-3-phosphoglycerol (PG) head groups and sphingomyelin. Such negatively charged membrane compositions with sphingomyelin act as a simple model for the plasma membrane. The interactions with both bilayers are probed using long unbiased simulations as well as umbrella sampling. In addition, the effect of anchoring the peptides to the membrane with a maleimide-containing lipid anchor^17^ is investigated. As a proof of principle, we also experimentally explore the adsorption of the peptides to giant unilamellar vesicles^42^ as a model system, using the same membrane composition as in the simulations.

## Results and Discussion

To compare the peptides’ interactions we have performed simulations of both peptides in solution, in the coiled-coil structure, and in close proximity to the neutral PC and negatively charged PG bilayer.

### Peptides in solution

In the simulations of the peptides in solution, as expected, the coiled-coil structure of the original three heptat coiled-coil *peptides E/K* remained stable, as expected, throughout the 600 ns simulation, with a root mean square deviation of ~2 Å from the native structure in the final frames. This agrees with experimental expectations^15,43^ which find a conformational stability of 9.6 kcal/mol or 40.2 kJ/mol, and a previous simulation study^44^. The individual *peptide K* in solution was observed to remain partially helical in two out of three different 600 ns simulation runs, and unfold in the third simulation. In contrast, *peptide E* adopted a random coil structure. These results are also consistent with CD experiments^17,45^, which indicate that *peptide K* remains at least partially folded, while no indication for alpha helical structure is found for *peptide E*. However, more recent measurements indicate substantial unfolding of both peptides^21^.

### Peptide-bilayer interactions

To understand how the peptides are affected by their interactions with the lipid bilayer, we performed simulations of each of the two peptides in the vicinity of lipid bilayers with two different compositions corresponding to compositions popularly used in two different fusion assays: a neural PC bilayer, which is rich in dioleoylphosphatidylcholine (DOPC), and a negatively charged PG bilayer containing dioleoylphosphatidylglycerol (DOPG). The helical peptides were simulated with a random initial orientation close to the bilayers. However, no interactions of *peptide E* and only few interactions of *peptide K* with the neutral PC bilayer could be observed within the 600 ns timescale of the simulations indicating that there are some significant repulsive interactions between the peptides and lipid head-groups. The conformations of the peptides in these simulations are summarized in Table 1 and the Supporting Information (SI).

**Table 1:** Characterization of helicity values of *peptides K* and *E* in solution and close to the PC and PG bilayers, data shown for three different unbiased simulation runs.

System	Helicity (%)*	Average COM distance (nm)^§^
*K/E* coiled-coil	95\pm ± 2, 98\pm ± 3, 95\pm ± 2	n.a.
*K* in solution	0, 57 ± 5, 43 ± 5	n.a.
*E* in solution	0, 0, 0	n.a.
*K* with PC bilayer	10 ± 4, 67 ± 4, 76 ± 5	4.2 ± 0.4, >5, >5
*E* with PC bilayer	19 ± 0, 19 ± 5, 43 ± 4	>5, >5, >5 (No interaction with the bilayer)
*LPK* in PC bilayer	57 ± 5, 38 ± 5, 81 ± 5	4.5 ± 0.7, 4.9 ± 0.6, 4.1 ± 0.3
*LPE* in PC bilayer	81 ± 5, 33 ± 5, 14 ± 5	3.6 ± 0.2, 3.4 ± 0.3, 3.7 ± 0.2
*K* with PG bilayer	76 ± 5, 76 ± 4, 57 ± 9, 81 ± 5^#^	3.1 ± 0.1, 2.8 ± 0.1, 3.4 ± 0.3, 2.8 ± 0.2^#^
*E* with PG bilayer	76 ± 4, 67 ± 4, 0, 76 ± 4^#^	2.9 ± 0.1, 3.0 ± 0.1, 3.4 ± 0.2, 2.7 ± 0.1^#^
*LPK* in PG bilayer	52 ± 5, 81 ± 5, 71 ± 5, 57 ± 4^#^	2.8 ± 0.2, 2.8 ± 0.1, 3.1 ± 0.1, 2.8 ± 0.2^#^
*LPE* in PG bilayer	67 ± 9, 71 ± 5, 62 ± 5, 33 ± 5^#^	2.9 ± 0.1, 3.1 ± 0.1, 2.9 ± 0.1, 2.9 ± 0.2^#^

*Helicity value at the end of the simulation reported only for the three heptat recognition sequence of the peptide.

^§^Distance between the center of mass (COM) of the peptide and the midplane of the bilayer.

^#^Simulation data for a bigger bilayer.

This behavior of *peptide E* agrees well with surface pressure measurements on monolayer using the Langmuir film balance technique^36^ and CD experiments^25,37^, which observed no interaction of *peptide E* with the neutral bilayer. The behavior of *peptide K* on the other hand is somewhat different from what would be expected based on both experiments^25,36,37^ and previous simulation studies^35,38,39^ that predict embedding of *peptide K* in the bilayer. There are different possible explanations for these differences. Most likely, the strongest effect comes from the steric repulsion of the large PC head groups, and the abundance of large and positively charged LYS side chains in the peptide which are repelled by the positive ${{\rm{NH}}}_{3}^{+}$ and ${\rm{N}}{({{\rm{CH}}}_{3})}_{3}^{+}$. These may create a large energy barrier for peptide embedding. Another difference from previous simulation studies is the presence of the glycine linker residues and CYS at the C-terminal of the peptide. In the following, we further investigate the influence of these different factors using umbrella sampling simulations, to overcome some of the sampling limitations.

#### Umbrella sampling simulations

**Figure 2:**
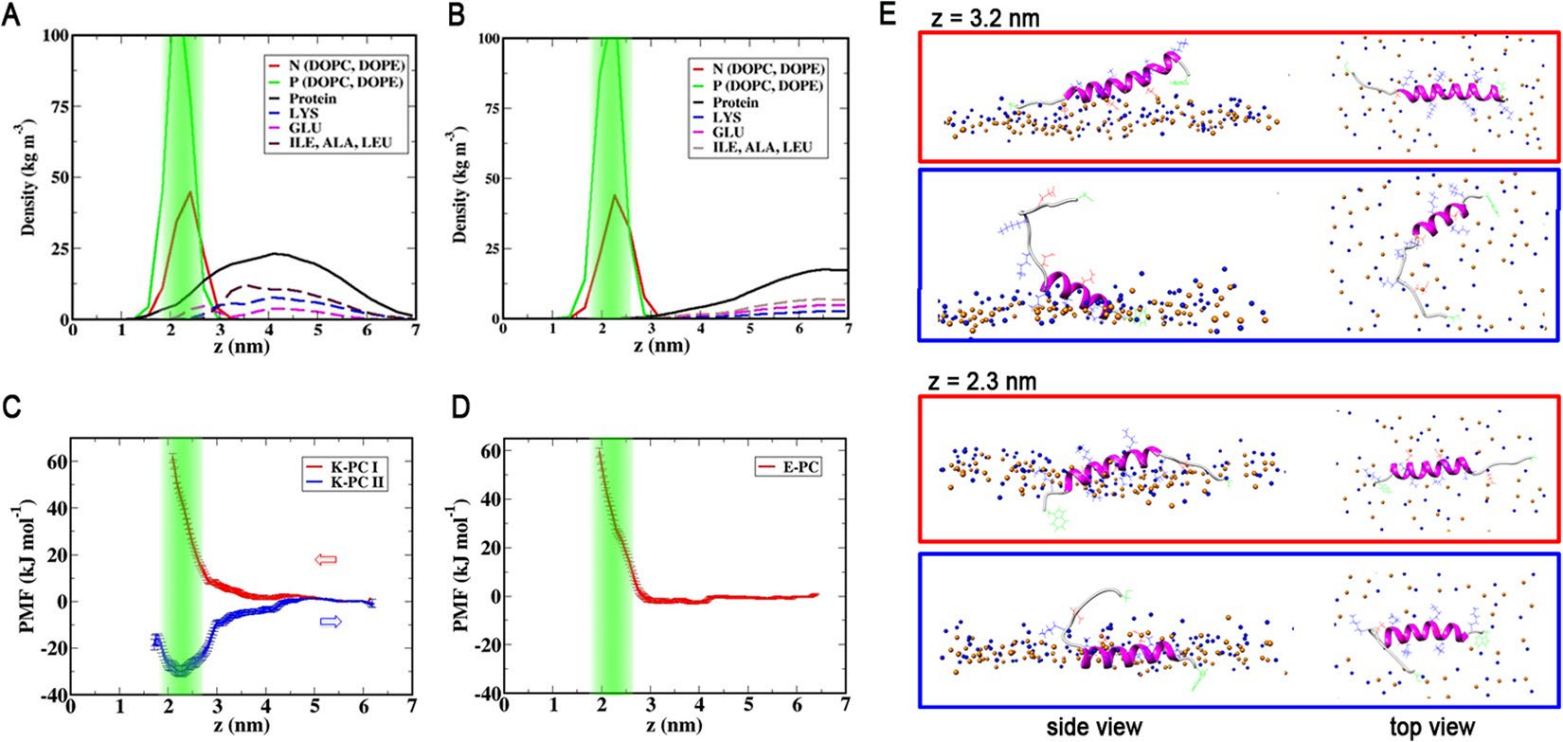
(**A**,**B**) Density distributions of lipid head-groups as well as charged and hydrophobic residues of the peptides: (**A**) for *peptide K* in the simulation, in which the peptide adsorbs to the bilayer; (**B**) for *peptide E*, which does not interact with the bilayer. The position of the phosphates is highlighted in green. (**C**,**D**) Umbrella sampling PMF profiles (red) for (**C**) *peptide K* and (**D**) *peptide E* as a function of the distance *z* from the bilayer midplane. The green area marks the positions of the phosphates in the lipid head-groups. In (**C**) the blue curve represents a second PMF generated by pulling an equilibrated helical *peptide K* from its position at the head tail interface. (**E**) Simulation Snapshots for two values of *z* from the two distinct PMF profiles in (**C**). Lipid head-groups are shown in blue (N) and orange (P), the peptide is drawn in magenta (helical) and light gray (coil), Side chains are drawn as blue (LYS), red (GLU) and green (TRP, CYS) lines.

To be able to better quantify the peptide membrane interactions, we have performed different umbrella sampling simulations to obtain the PMF experienced by the peptide as a function of the distance *z* from the bilayer midplane. The corresponding PMF profiles are shown in Fig. 2.

In a first set of umbrella sampling simulations, the initial states for each umbrella window were generated, by slowly moving the unrestrained peptides from the solvent towards the bilayers. The PMF profiles for both peptides steeply increase close to the bilayer surface, where the peptides come into contact with the bulky head groups. Neither profile shows a stable minimum below the head-group region.

These general features of the PMF profiles remain at least partly at odds with previous experimental observations and computational studies, as discussed above. It has been found previously, that long time scale transitions and energy barriers along orthogonal reaction coordinates can greatly affect the PMFs obtained with similar reaction coordinates close to a lipid bilayer^46,47^. To investigate the importance of this effect, an additional PMF was calculated, in which the helical peptide was pulled from an equilibrated position at the head tail interface into solution. For *peptide K*, this profile, also shown in blue in Fig. 2C, shows a distinct minimum with a depth of −30 kJ/mole (−12 *k*_*B*_*T*) at *z* = *2*.*2* nm, which corresponds to the head tail interface. In contrast to *peptide K*, equilibrating a helical *Peptide E* in the membrane was not possible and lead to rapid unfolding and expulsion of the peptide in all cases, so that a corresponding PMF profile for *peptide E* could not be obtained. This behavior indicates that for *peptide E* the interactions with the bilayer are unfavorable in either case.

The PMF should be an equilibrium quantity, and should in principle be independent of how the initial states are generated. The strong hysteresis observed here, shows that other relevant slow processes, which are not well captured by the simple reaction coordinate, play a significant role for the adsorption of *peptide K*. For instance, the second PMF profile does not show a high energy region corresponding to the peptide interacting with the lipid head-groups. Both profiles remain more or less flat to approximately z = 3 nm, and the simulation snapshots shown in Fig. 2E indicate that this corresponds to the peptide moving from solution towards the bilayer until it is aligned at the top of the head-group region. To move closer towards the bilayer midplane, the peptide has to pass the bulky head-groups. In the simulations corresponding to the blue PMF on the other hand, the initial bilayer structure is equilibrated around the peptide, so that there is a fitting void present in the bulky PC head groups, clearly visible in the top-view snapshot at z = 2.3 nm shown in Fig. 2E. The peptide is aligned parallel to the bilayer with the hydrophobic side chains embedded and the LYS side chains spread out between the head-groups. The N terminal TRP sidechain is embedded the deepest. For the red PMF, the TRP sidechain is also embedded deepest, however the rest of the peptide is aligned forms an angle with the bilayer. Several head-groups are located very close to or underneath the peptide, leading to the strong repulsion seen in the PMF. Another factor that should in principle contribute to the PMF profile is the peptides’ secondary structure, as the peptides will unfold in solution. In the comparatively short umbrella simulations, the peptides remain predominantly helical, similar as in the restrained CG simulations. A free energy gain for unfolding the peptide in solution would reduce the free energy in solution, so that the value of the minimum free energy, Δ*G* = −12 *k*_*B*_*T* from the second PMF represents an upper limit for the membrane partitioning free energy. In principle, peptide secondary structure could be addressed by combining umbrella sampling with replica exchange or metadynamics, however, given the large effect of the lipid degrees of freedom, we will instead compare free profiles generated following the same procedure, while keeping in mind that the exact numerical values contain a certain bias.

This upper limit Δ*G* = −12 *k*_*B*_*T* would predict a partition coefficient, ${K}_{p} \sim 150\times {10}^{3}{M}^{-1}$ using the molar volume of DOPC obtained from the simulations as 1.0418 l. This partition coefficient is about 25 times larger, than the experimental estimate ${K}_{p} \sim 6.2\times {10}^{3}{M}^{-1}$ predicted from fluorescence measurements.

A PMF profile was also calculated based on the center of mass distance distribution for adsorption events observed in the CG simulations^38^. The free energy minimum of 7,7 kJ/mole in that profile would correspond to a value of *K*_*p*_ that is two orders of magnitude smaller. Taken together, these data indicate that the free energy difference obtained by pulling the peptide out of the bilayer shows a reasonable agreement with experimental data, is likely to be closer to the “real” picture. Even this upper limit for the free energy difference of Δ*G* = 30 kJ/mol, is still about 10 kJ/mol smaller than the energy gain of 40 kJ/mol for coiled-coil formation reported for the *peptides E/K* system^15,43^. Thus, if free *peptide E* is available for the coiled-coil formation, *peptide K* is at least 50 times more likely to form a coiled-coil than to bind to the membrane.

To test for contributions of the additional flexible residues at the C terminal of the peptide to the bilayer interactions and to the observed differences, further PMFs using only the original peptide recognition sequences were calculated. The resulting profiles are virtually identical with those shown in Fig. 2. Therefore, we can conclude, that the extra residues do not significantly affect the peptide-bilayer interactions for *z* values outside the head-group region.

**Figure 3:**
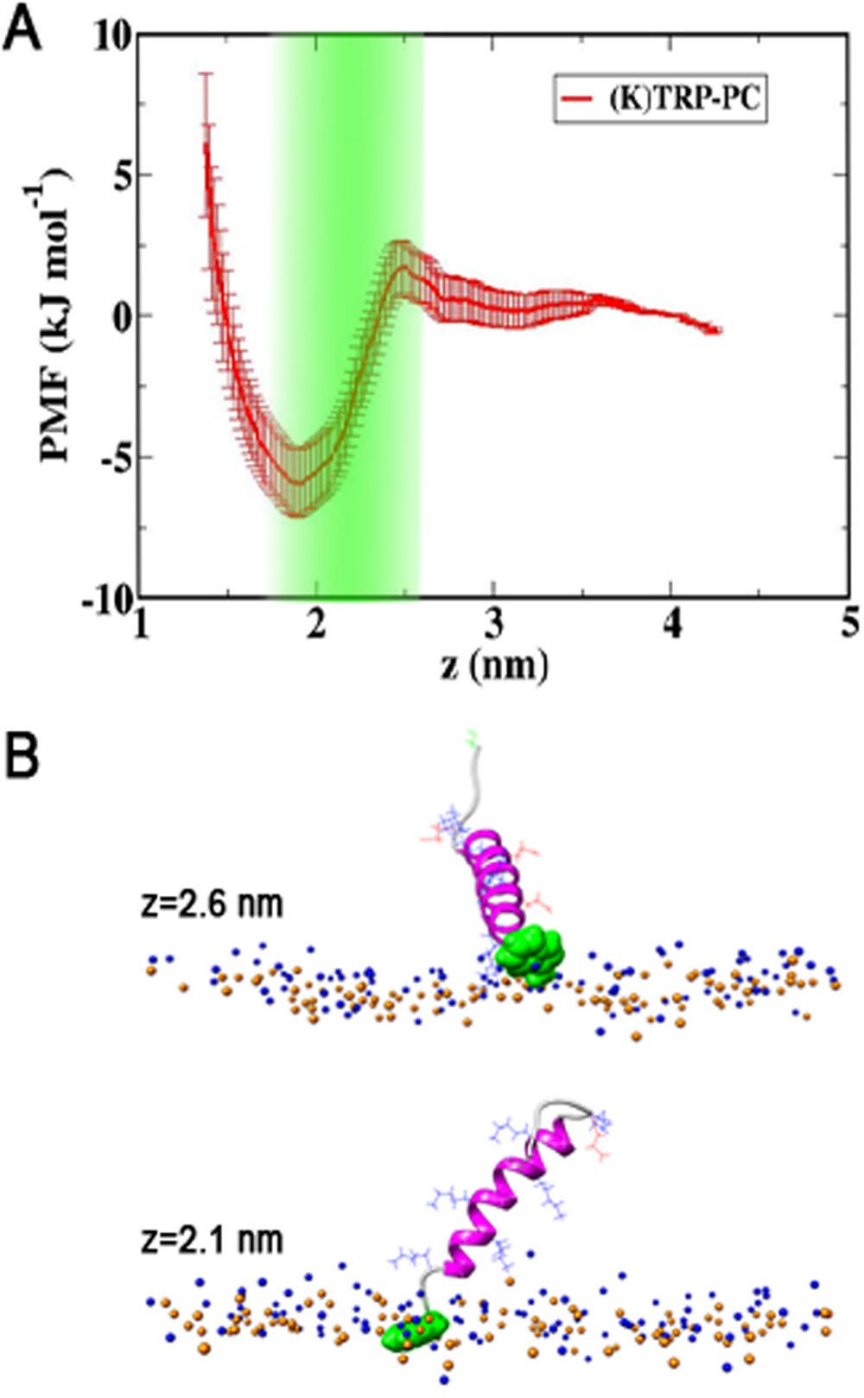
(**A**) PMF for TRP residue in *peptide K* as a function of the distance *z* from the midplane of the PC bilayer. (**B**) Simulation Snapshots for two values of *z*. Lipid head-groups are shown in blue (N) and orange (P), the peptide is drawn in magenta (helical) and light gray (coil). The TRP is shown in green, other side chains are drawn as blue (LYS), red (GLU) lines.

Finally, it has been observed in previous studies of helical antimicrobial peptides^48–51^ as well as for *LPE* (described below), that aromatic residues, in this case the tryptophan (W) residue of the linker, show the greatest tendency to interact with the membrane and embed in the lipid tail region. Experiments by Rabe *et al*.^25^ have predicted absorption of *peptide K* in the PC bilayer based on tryptophan quenching measurements. To exclude the speculation that the partitioning results are biased by the stronger membrane affinity of the TRP residue, we have calculated the PMF experienced by the center-of-mass of indole ring of the TRP residue at the N terminal of *peptide K*. The profile, shown in Fig. 3, has a shallow minimum of −6 kJ/mol at *z* ≈ 1.9 nm, which is in the lipid tail region, just below the head tail interface, even though the rest of the peptide remains in solution. However, the minimum is not deep and the partitioning of the peptides that would be predicted by this, is negligible compared to the value measured by the fluorescence quenching, and therefore unlikely to affect the results.

### Effect of bilayer composition: peptide-PG interactions

The composition of the neutral bilayer (DOPC:DOPE:Chol 2:1:1), which will be referred to as ‘PC’ bilayer, corresponds to the most common lipid composition used to study vesicle fusion and peptide membrane interactions of the fusion *peptides E/K* system in experiments^17,25,36,37^ and simulations^35,38,39^. However, recent experiments have also used *LPE* substituted in a lipid bilayer containing charged DOPG lipids to show docking and fusion^41^. The bulky PC head-groups represent a significant contribution to the energy-barrier for peptide insertion, so that a smaller fraction of PC head-groups in this composition may lead to a reduction of this barrier, while the presence of charged lipids is likely to affect interactions with the charged side chains.

**Figure 4:**
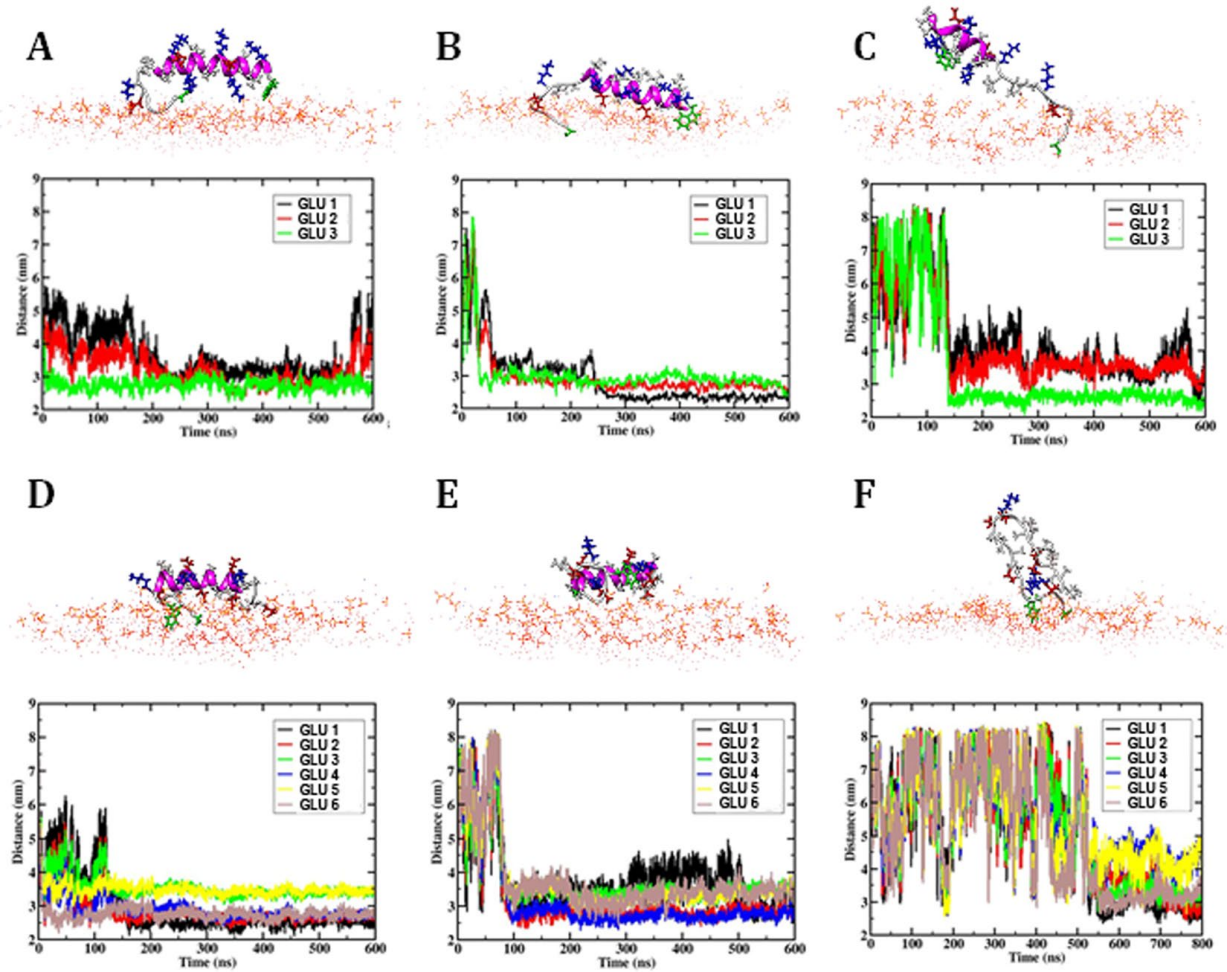
Simulation snapshots of the peptide orientation at the head-group water interface and time evolution of the distances of the GLU residues from the bilayer midplane for three independent simulations of unanchored peptide close to the PG bilayer: (**A**–**C**) *peptide K* and (**D**–**F**) *peptide E*. In the simulation snapshots, DOPG and sphingomyelin head groups are shown as orange lines, the peptide backbone is shown in magenta (helical part) and light grey (random coil); side chains are drawn in stick representation in red (GLU), blue (LYS) and white(hydrophobic); TRP and CYS side chains are shown in Green. The GLU residues are labeled 1–3 in *peptide K* (**A**–**C**) and 1–6 in *peptide E* (**D**–**F**) according to their position in the sequence, i.e. starting from the N terminus.

In general, it has emerged that lipid composition may significantly affect membrane peptide interaction as well as peptide structure. For example the presence of charged PG head-groups can induce structural changes in cell penetrating peptides from unstructured to beta sheet or alpha helical structure^52,53^. Therefore here, we have modeled the interactions of the two fusion *peptides K* and *E* with a bilayer of a second composition, containing DOPG, sphingomyelin and cholesterol in a ratio [6:3:1] chosen to represent that of the liquid disordered phase^40,41,54^ and denoted in the following as the ‘PG’ bilayer. Indeed, the peptide bilayer interactions observed for this composition are shown to be significantly enhanced. In the unrestrained simulations, both peptides adsorb at the bilayer water interface and retain their helicity to a larger extend as summarized in Fig. 4 and Table 1.

The initial adsorption process proceeds comparatively quickly, guided by the longer range electrostatic interactions. This is reflected for instance in the distance vs time curves for the GLU residues, which are shown in Fig. 4, together with the simulation snapshots. After adsorption, peptide mobility is reduced due to interactions with lipid head groups and the conformation does not significantly change anymore, so that the final state is predominantly determined by the adsorption process. More details for the individual simulation runs can be found in the SI. The GLU side chains of both peptides interact with the Ca^2+^ ions in the head-group region acting as a bridge to the ${{\rm{PO}}}_{4}^{-}$ groups. The CYS and TRP residues in the linker sequence which interact strongly with both membranes in *LPK* and *LPE* do not interact with the membrane before the charged residues do. In *peptide K* the extended peptide sequence and the N-terminal TRP interact most strongly with the bilayer, reflected for instance in a visible second step after 250 ns in the GLU distance curves in Fig. 4B, where the N terminal TRP residue inserts into the bilayer.

**Figure 5:**
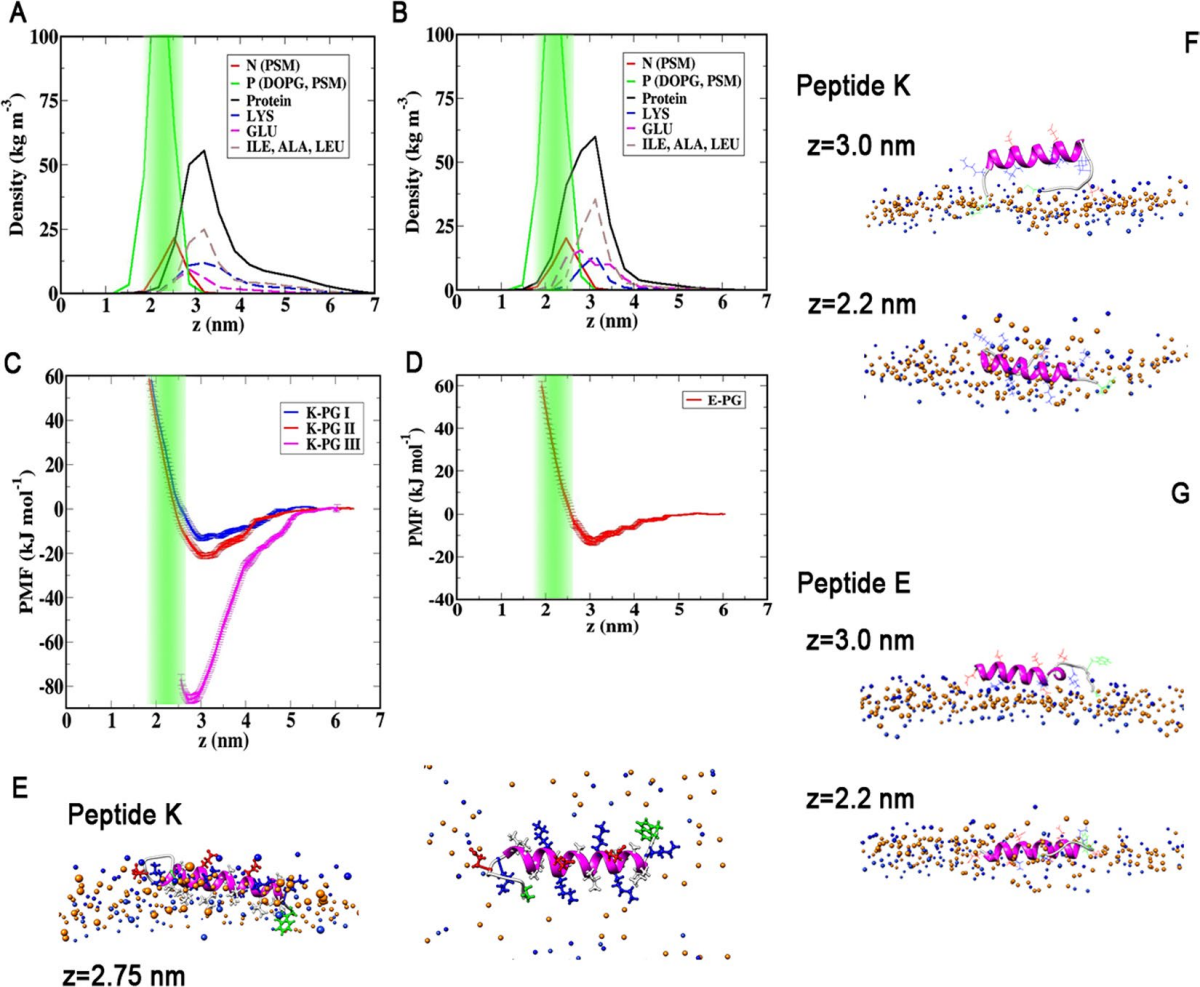
(**A**,**B**) Density distributions of lipid head-groups, charged and hydrophobic residues for the PG bilayer: (**A**) for *peptide K* and (**B**) for *peptide E*. The position of the phosphates is highlighted in green. (**C**,**D**) umbrella sampling PMF profiles for (**C**) *peptide K* and (**D**) *peptide E* as a function of the distance *z* from the bilayer midplane. The different color profiles in (**C**) correspond to simulations with the hydrophobic tails (blue) and LYS side chains (red) facing the bilayer, and for initial states generated from pulling the peptide out of the bilayer (magenta). The green area marks the positions of the phosphates in the lipid head-groups. Panels **(E–G)** show snapshots of the bilayer peptide systems for different values of *z*. The snapshots in (**E**) correspond to the deep minimum in the pulling-out profile.

As for the PC bilayer, several PMF profiles as a function of the distance *z* from the bilayer midplane were calculated for the PG bilayer. When the starting configurations are generated from pulling the peptide into the bilayer, the PMF profiles shown in Fig. 5, again show a steep energy increase inside the bilayer. However, for the charged bilayer, both PMF profiles also possess a shallow minimum at the head-group water interface, which agrees with the observation that the helical peptides adsorb there in the unbiased simulations.

For the PMF profiles for *peptide K* facing the bilayer in different orientations, the depth of the minimum varies between −13.2 kJ/mole (−5.3 *k*_*B*_*T*) and −21.4 kJ/mole (−8.6 *k*_*B*_*T*) for orientations with the hydrophobic side chains and LYS side chains facing the bilayer, respectively. For *peptide E*, the corresponding minimum has a free energy of only −13.0 kJ/mole (−5.2 *k*_*B*_*T*). For all PMF profiles, the minima are located at the head-water interface rather than below the head-groups. The deeper minimum with LYS facing the bilayer reflects the favorable electrostatic interactions between positive charges and negatively charged PG head-groups and the reduced steric interactions of the LYS side chains, which are now aligned with the bilayer normal.

As for the PC bilayer another PMF profile was calculated, initial states for which were generated by moving the peptide out of the bilayer. A similar equilibrium conformation on the membrane with the hydrophobic side chains facing the membrane interior can be expected, and indeed a similar conformation, including also the lysine ‘snorkle’ effect was observed in an unbiased simulation using a larger bilayer patch (see SI) which was used as the starting conformation for an equilibrated membrane bound state. As expected, the corresponding PMF profile shows a deep minimum of 86 kJ/mol (35 *k*_*B*_*T*). As before, the strong hysteresis in the profiles means that this value represents an upper limit for the binding free energy. However, a direct comparison between the profiles for the neutral bilayer, indicate a significantly stronger interaction for *peptide K* with the charged PG bilayer.

### Experimental comparison of peptide-membrane interactions for the two bilayer compositions

**Figure 6:**
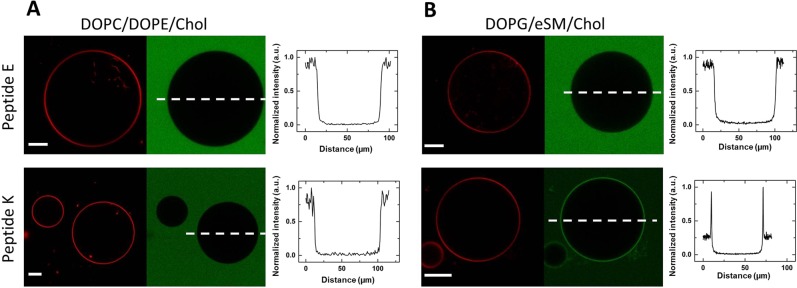
Interaction of *peptides E and K* with GUVs made of (**A**) DOPC/DOPE/Chol or (**B**) DOPG/eSM/Chol. The red and the green channels indicate the respective signal from the membrane of the GUVs and the labeled peptides. The intensity line profiles show the distribution of the peptides along the dashed lines indicated on the images. The concentration of the *peptides E* and *K* is 7 μM and 3.25 μM, accordingly. The scale bars correspond to 20 μm.

Experimental control data of labeled *peptide K* interacting with phase separated giant vesicles containing PG show, that *peptide K* adsorbs to the membrane^41^. To confirm the increased interactions of *peptide K*, giant unilamellar vesicles (GUVs) with compositions corresponding to those used in the simulations were prepared and incubated with fluorescently labeled *peptides E* and *K*. The confocal microscopy images and extracted intensity line profiles in Fig. 6 show a co-localization of *peptide K* with the PG bilayer (even at lower peptide concentration), whereas no enrichment was measurable for the neutral bilayer, nor for *peptide E* at either bilayer composition and higher peptide concentration. These results confirm that the membrane interactions of *peptide K* are significantly enhanced by the presence of the charged PG lipids in the membrane. Quantitative comparison between the experimental and simulation data was not done considering the difference of the solutions bathing the bilayers (note that the peptide binding to the GUVs was examined in the absence of calcium ions as they destabilize charged GUVs^55^.

### Size dependence: larger bilayer patch

It is possible that the finite size of the simulation box has an effect on peptide insertion, as the areas of the two monolayers are coupled. In an unrestrained simulation of the adsorption process of the peptides to a PG bilayer patch with twice as much area, both peptides adsorb slightly deeper (See SI and Table 1). However, large differences are also observed between individual runs for the same bilayer. To address this further, PMF profiles for the two bilayer sizes were compared. The profiles from the two bilayer sizes have minima at the same value of z and the depth of the free energy minima varies less between the bilayer sizes than the observed differences due to other factors. The corresponding data can be found in Fig. S3 in the SI. Thus, using a larger bilayer patch for all simulations does not promise a great improvement, compared to other computational approaches.

### Effect of lipid anchor in the PC bilayer

**Figure 7:**
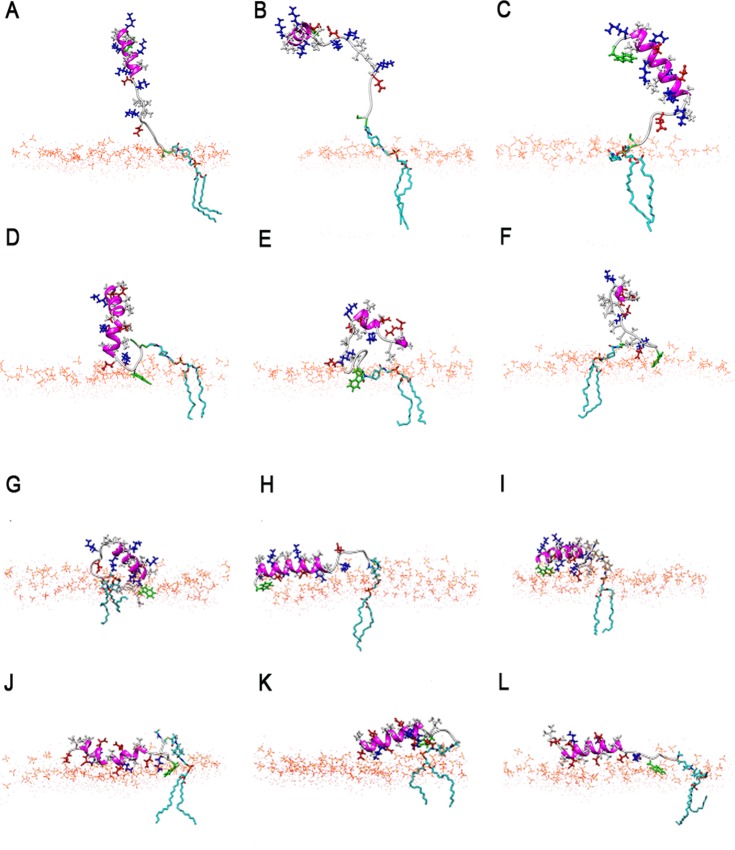
Snapshots from simulations of the lipid-anchored peptides: (**A**–**C**) *LPK* anchored to the PC bilayer, (**D**–**F**) *LPE* anchored to the PC bilayer, (**G**–**I**) *LPK* anchored to the PG bilayer, (**J**–**L**) *LPE* anchored to the PG bilayer. The peptide backbone is shown in magenta (helical parts) and light grey (unstructured), side chains are drawn in red = negative, blue = positive, white = hydrophobic residues, TRP and CYS side chains are highlighted in green; lipid head groups are shown in orange, lipid tails are only shown for the peptide anchor.

Experiments using different lipid anchors^22^ and different spacer lengths^21^ have indicated, that the anchor properties and enforced vicinity to the membrane may affect the peptide structure and even fusion efficiency. We have simulated lipid-anchored peptides *LPK* and *LPE*, in which the peptides are attached to a maleimide-containing lipid anchor (Fig. 1B). Successful docking and fusion events have been observed using these molecules^17^. In unbiased simulations, the lipid -anchored peptide *LPK* behaves similar to the free *peptide K* close to the PC bilayer: the helical structure of the peptide is not retained in the proximity of the membrane, as summarized in Fig. 7A–C and Table 1. Overall, CD spectroscopy experiments^25,37^ have found the helicity for the lipid -anchored peptide *LPK* to be similar to that of free *peptide K* in solution with lipid vesicles, for most lipid anchors. The measured values are in the range 44–48%, which is in a good agreement with those observed here. The C-terminal GLU and LYS side chains remain at the head-group interface for most of the simulation. The C-terminal GLU side chain forms two hydrogen bonds with the ${{\rm{NH}}}_{3}^{+}$ group of DOPE; the C-terminal LYS forms 2–3 hydrogen bonds with ${{\rm{PO}}}_{4}^{-}$ and one hydrogen bond with the choline group before peptide unfolding. Despite these interactions, the unfolding of the helix proceeds from the C-terminal end, which is attached to the linker and thus forced to remain close to the bilayer in all conformations.

The anchored peptide *LPE* also largely remains exposed to the solvent, but – in contrast to the free *peptide E* -is now restrained in the vicinity of the bilayer. In three different simulations, *LPE* is found to be almost helical, partially helical (with ~38% helicity) and completely unfolded (Fig. 7D–F). The TRP (W) in the GWGGGC-linker sequence inserts most deeply into the lipid tail region. This behavior agrees with the expectation that membrane insertion often starts with aromatic amino acids^48–51^. Because the TRP is located close to the main peptide sequence, this has the effect of an effectively much shorter linker. The effect of the TRP insertion is noticeable especially for the deeper insertion of the C-terminal GLU and LYS residues. Accordingly, more contacts with the lipid head-groups are observed, especially for the charged amino acids close to the C-terminal. On average, the C-terminal GLU side chain forms 12–15 contacts with ${\rm{N}}{({{\rm{CH}}}_{3})}_{3}^{+}$, and 4–8 contacts with ${{\rm{NH}}}_{3}^{+}$. Overall, peptide orientations with the negatively charged GLU side chains facing the bilayer and interacting with the ${\rm{N}}{({{\rm{CH}}}_{3})}_{3}^{+}$ and ${{\rm{NH}}}_{3}^{+}$ groups are favored, leading to 10–15 contacts and 4–6 contacts of the other GLU residues with $\,N{({{\rm{CH}}}_{3})}_{3}^{+}$ and ${{\rm{NH}}}_{3}^{+}$, respectively. Through these interactions, the enforced vicinity to the bilayer, here has a stabilizing effect on the helical structure, and at least significantly slows down unfolding compared to the peptide in solution. Experimentally, a stabilizing effect of the enforced vicinity of the bilayer to *LPE* has been reported^25^, which has been attributed to the formation of homo-dimers as a result of the greater number of peptides close to the bilayer^25,37^, because the fluorescence of TRP is not quenched by the bilayer. The electrostatic interactions between the GLU residues along the side of the peptide with the positive head-group surface observed here however, suggests that the membrane can also contribute to the helix stabilization.

### Effect of lipid anchor in the PG bilayer

Since both peptides interact spontaneously with the bilayer, the lipid-anchored peptides behave very similarly to the untethered peptides. Peptide *LPK* adsorbs quickly at the head-water interface, with the helix axis oriented parallel to the bilayer plane, and largely remains helical. The largest number of hydrogen bonds between charged side chains and head-groups, on the order of 10, is formed for the peptide retaining the most helicity. In all three simulations, the positively charged LYS side chains face the bilayer, as would be expected from electrostatics and the deepest minimum in the PMFs for that orientation. Similarly, lipid-anchored peptide *LPE* remains adsorbed on the bilayer surface with the helix axis at an angle ~90° from bilayer normal in all three simulations. The peptide adsorbs within less than 100 ns starting from the C terminal in all cases. Adsorption starts with the Ca^2+^ mediated interaction of the C-terminal GLU, and the peptide aligns with the GLU side chains facing the bilayer. The GLU and LYS side chains form several hydrogen bonds with the DOPG head-groups. A similar extent of unfolding as for *LPK* is observed.

## Conclusions

In summary, we used atomistic MD simulation to study the interactions of the two synthetic fusion *peptides K* and *E* and their lipid-anchored counterparts *LPK* and *LPE*, shown in Fig. 1, with two different bilayer compositions, one neutral (PC) and one negatively charged (PG).

In long, unbiased MD simulations both *peptides K* and *E* experience a long-range repulsion from the zwitterionic PC and PE head-groups and behave similarly as in the absence of a bilayer, unfolding to a large extend. Umbrella sampling simulations were used to overcome the repulsion of the lipid head groups. The PMF profiles in Fig. 2 reflect the strong repulsion, showing a rapid increase of free energy, as the peptide approaches the head-groups. A large hysteresis effect is found when pulling the helical *peptide K* out of an equilibrated bilayer. The PMF calculated for these simulations (Fig. 2C) shows a stable adsorbed state of *peptide K* at the head-tail interface, with a binding free energy of about −12 *k*_*B*_*T*. Although this value overestimates the partitioning of the peptide into the membrane compared to experimental results, it is closer to the experimental value than previous predictions and can be viewed as an upper bound for the binding free energy. The latter free energy is expected to be reduced both by the effects of the hysteresis, and the energy of peptide unfolding in solution which is not expected to fully equilibrate during the umbrella sampling simulations. Nevertheless, even using this maximum value of Δ*G* = −12 *k*_*B*_*T* the partitioning free energy is still by about 10 kJ/mol smaller than that of coiled-coil formation^15,43^. Based on these values, if *peptide E* is available, a *peptide K* would be 50 times more likely to form a coiled-coil than to bind to the membrane, and more so if the experimentally estimated values are used. As typically only 1 in about 100 lipids is *LPE* this does not preclude membrane interactions of *peptide K*. It is in principle also possible, that the whole coiled-coil interacts with the membrane, however due to the shielding of hydrophobic residues, this is highly unexpected, and TRP quenching of the peptide complex *K/E* with TRP labeled *peptide E*, show no increased interactions of* peptide E* with the bilayer^25^. Therefore, membrane interactions of the coiled-coil have not been explicitly addressed here. Although the TRP residue in *peptide K* was found to interact most strongly with the bilayer, the free energy minimum in the PMF profile for this residue (Fig. 3) when the rest of the peptide stays in solution is only of the order of 2 *k*_*B*_*T*. This value is small compared to the partitioning measured based on fluorescence quenching of TRP, which implies a binding free energy of *8.7 k*_*B*_*T*, so that the bilayer affinity of TRP is not likely to introduce any appreciable bias to the TRP quenching results.

In comparison, the interactions of the fusion peptides with the PG-containing bilayer are found to be significantly enhanced by the presence of the charged DOPG lipids. Both peptides adsorb onto the bilayer in unbiased simulations as summarized in the snapshots and the peptide-bilayer distance evolution plots shown in Fig. 4. The helical secondary structure of the peptides is stabilized by these interactions with the bilayer. The corresponding PMF profiles shown in Fig. 5 all exhibit free energy minima at the head-group water interface, consistent with the spontaneous adsorption. Comparing the minima for pulling *peptide K* out of the bilayer shows that the binding free energy to the PG-containing bilayer is much larger than for the PC bilayer. This simulation result is confirmed experimentally by the adsorption of fluorescently labeled peptides to GUVs of the respective composition, summarized in Fig. 6. Therefore, if peptide-membrane interactions play a role in the fusion mechanism of this SNARE mimetic system, as has been suggested previously, the presence of charged PG lipids in the bilayer should enhance the role of these interactions and may thus affect the fusion efficiency.

The lipid-anchored peptides *LPK* and *LPE* are forced to remain in close proximity to the bilayer, by the attachment to a lipid anchor. For the neutral PC bilayer, this has an effect mainly on *LPE*, which contains six glutamic acid residues that interact with the choline and amine groups of the bilayer’s head-group region (red side chains in Fig. 7D–F). Through these interactions *LPE* remains more structured than *peptide E* in solution. *Peptide K* and the lipid anchored *LPK* contain only three GLU side chains. These side chains are located on the face opposite to the hydrophobic residues, making interactions with the bilayer less favorable. For the PG bilayer the lipid anchor has no observable effect, because the peptides bind to the bilayer already in the absence of the lipid-anchor.

## Methods

To characterize the peptide-bilayer interactions, we have simulated the following systems: (i) single peptides and a coiled-coil in solution; (ii) peptides in the vicinity of the bilayer; (iii) lipid-anchored peptides at the bilayer; and (iv) umbrella sampling simulations for all peptide-bilayer combinations.

### System setup and initial structures

#### Peptides

Initial helical structures of the peptides were obtained from the PDB database (1U0I)^43^. For systems (ii–iv), peptides with an extended amino acid sequence, Ac-WG-(KIAALKE)_3_-GGGGC-N(H)(CH_3_) for *Peptide K* and Ac-(EIAALEK)_3_-GWGGGC-N (H)(CH_3_) for *Peptide E*, as used in experiments conducted by Pähler *et al*.^17^, were prepared for the simulations (Fig. 1). The helicity percentage values are calculated with respect to the helical coiled-coil recognition unit only. The additional residues were attached to the termini in a random coil conformation, as predicted from homology modeling using the Mobyle@RPBS server^56^. The peptide N and C termini were capped with neutral acetyl and methyl amide residues, respectively. For simulations of the peptides in solution, ~10000 water molecules were added to the system for *peptides E* and *K*; ~17000 water molecules were added for the coiled-coil structure.

To create the lipid-anchored peptide structures, denoted ‘*LPK*’ and ‘*LPE*’, the hydrogen attached to sulfur in the C terminal CYS was removed. Similarly, a DOPC lipid was modified to create the lipid anchor, by replacing the three methyl groups in the choline by one hydrogen atom and a bond to connect to the linker molecule shown in Fig. 1. The other end of the linker was attached to the modified CYS residue.

#### Lipid bilayers

Two bilayer compositions were modeled; a neutral membrane consisting of DOPC, DOPE and cholesterol in a ratio 2:1:1 and a negatively charged bilayer containing DOPG, cholesterol and sphingomyelin in a ratio 6:1:3. Initial bilayer structures were generated with the CHARMM-GUI membrane builder^57–59^. The final bilayer systems consisted of 128 DOPC, 64 DOPE and 64 cholesterol molecules solvated in ~22000 water molecules for the PC bilayer, and 154 DOPG, 78 the sphingomyelin and 26 cholesterol molecules solvated in ~26600 water molecules for the PG bilayer. For the PG bilayer, a larger bilayer patch with 308 DOPG, 154 sphingomyelin and 50 cholesterol molecules was also constructed.

#### Peptide bilayer systems

For systems (ii), the peptide was placed approximately 3 nm above the pre-equilibrated bilayer surface in a random orientation. Systems (iii) with membrane-anchored peptides were prepared by replacing one of the DOPC or DOPG molecules with the lipid anchor part of the *LPK* or *LPE* molecule. The structures were solvated with approximately 6 nm of water layer; water molecules in the lipid tail region were removed. For the PC bilayer, Ca^2+^ and Cl^−^ ions were added to produce a concentration of 50 mM CaCl_2_ corresponding to experimental conditions used in fusion assays. For the negatively charged PG bilayer, the Ca^2+^ counter ions already correspond to an ion concentration >50 mM, so that only counter ions were added. Three different initial structures with different initial peptide orientations were generated for each peptide-bilayer systems, resulting in a total of 8 × 3 initial structures. One additional structure with a bigger PG bilayer was also constructed for both *peptides K* and *E*; anchored and unanchored (4 × 1).

### Simulation parameters

The CHARMM36 force field^44,60^ was used for lipids and peptides, together with the TIP3P water model^61^. The missing bonded and LJ parameters for the lipid-anchor were generated using the SWISSPARAM server^62^. Partial charges for the modified CYS residue, the maleimide-containing lipid anchor and the modified DOPC lipid, were obtained in accordance with the CHARMM and CGenFF^63,64^ force field protocol from quantum chemical calculations with the MP2/6–31 G* and HF/6–31 G* levels of quantum chemical calculations, and by RESP fitting^65^ using the R.E.D. tools^66^.

### Simulation protocol

All simulations were performed with Gromacs 5.1.2^67^ in the NPT ensemble, using a 2 fs time step. The temperature was kept constant at 303.15 K by coupling the bilayer/protein and the solvent separately to the V-rescale thermostat^68^, using a time constant of 1 ps. The pressure was maintained at 1 bar in both the lateral and the normal direction with semi-isotropic pressure coupling to the Parrinello-Rahman barostat^69^, using a time constant of 5 ps and compressibility 4.5 × 10^−5^ bar^−1^. Van der Waals interactions were smoothly shifted to zero in the range between 1.0 nm to 1.2 nm. Long-range electrostatic interactions beyond the cut-off 1.2 nm were calculated using PME^70^. Bonds involving hydrogen atoms were constrained with the LINCS algorithm^71,72^, water molecules were kept rigid using SETTLE^73^. Center-of-mass motion removal was applied to the bilayer/protein and solvent groups separately. All peptide bilayer systems were simulated for 600 ns, unless stated otherwise. Visualization was done using VMD^74^ and UCSF-Chimera^75^.

### Umbrella sampling

To calculate the potential of mean force (PMF), umbrella sampling simulations were performed for all bilayer-peptide combinations. The distance along the bilayer normal of the peptide center-of-mass from the center-of-mass of the bilayer was chosen as the reaction coordinate *z*. The value *z* = 0 corresponds to the bilayer midplane. For each PMF profile, 40–45 umbrella windows between *z* = 1.7 nm and *z* = 6.0 nm were created, by slowly pulling the peptide from *z* = 6.0 nm to *z* = 1.7 nm with a force constant of 1000 kJ mol^−1^ nm^−2^ and selecting trajectory frames with the peptide position in the center of each window. Each conformation was run for 100 ns (unless stated otherwise) after a short equilibration. The last 50 ns of the trajectories were used to calculate the PMF profile with the Weighted Histogram Analysis Method (WHAM)^76,77^. Error analysis was done with the Bayesian bootstrapping method for complete histograms^78^.

### Experimental materials

The lipids 1,2-dioleoyl-sn-glycero-3-phosphocholine (DOPC); 1,2-dioleoyl-sn-glycero-3-phosphoethanolamine (DOPE); 1,2-dioleoyl-sn-glycero-3-phospho-(1′-rac-glycerol) (DOPG); Egg sphingomyelin (eSM); and cholesterol were purchased from Avanti Polar Lipids (US). Sucrose and D-glucose were obtained from Sigma Aldrich (St. Louis, MO). DOPE-ATTO 633 was purchased from Sigma-Aldrich (US) and the Oregon Green 488 maleimide (OG 488) ordered from Molecular Probes, part of Thermo Fisher Scientific (US). Tris, NaCl and HCl (37%) were from Roth (Germany). Chloroform was purchased from Merck (Germany). Dimethyl sulfoxide (DMSO) was from Sigma-Aldrich (US). The *E and K peptides* were kindly provided by Prof. Andreas Janshoff’s group at Georg August Universität, Göttingen.

### Vesicle preparation

Giant unilamellar vesicles (GUVs) were electroformed from 4 mM chloroform lipid solutions of either DOPC/DOPE/Chol in molar ratio (2:1:1) or DOPG/eSM/Chol in molar ratio (6:3:1). Both lipid mixtures contained 0.2 mol% DOPE-ATTO 633 fluorescently labeled lipid. The vesicles were formed following the procedure in^79^. Briefly, 16 µL of the lipid stocks were spread on a pair of indium tin oxide coated glasses and the latter were kept at room temperature in low pressure conditions for 2 hours to evaporate the organic solvent. The glasses were placed with their conductive sides facing each other, separated by a 2 mm thick Teflon spacer to form a chamber. The lipid films were hydrated with 100 mM sucrose solution, introduced in the swelling chamber. Using a function generator, an AC field (1.1 V, 10 Hz) was applied for 1–2 hours.

### Labelling the *E* and *K* peptides

The *E* and *K* *peptides* were labelled with OG 488 maleimide in a click reaction between the N-terminal thiol group of the peptides and the maleimide group of the dye. The OG 488 was dissolved in 2:3 ratio of Tris buffer (100 mM NaCl and 10 mM Tris adjusted to pH 7.5 with HCl) and DMSO to obtain a 2 mM solution. 1.25 mL of the latter was added to 3.75 mL of 100 µM *E* or *K* *peptide* dissolved in Tris buffer. The reaction was left stirring for 1 hour at room temperature. The labelled peptide was purified from the residual using size-exclusion chromatography performed on a PD-10 column (GE Healthcare, Great Britain), following the manufacture’s manual. Briefly, the column was equilibrated with 25 mL of 100 mM glucose solution prior to loading the sample. If the sample was less than 2.5 mL, glucose solution was added to adjust the total volume to 2.5 mL. After the sample was loaded into the column, 0.5 mL elution fractions were collected. 100 mM glucose solution was used for the elution. The fractions were screened via absorbance at 280 nm (detecting the aromatic amino residues of the peptides) and at 495 nm (detecting the OG 488).

### Vesicle imaging

The electroformed vesicles (in 100 mM sucrose solution) were harvested from the electroformation chamber and incubated for 10 min with the labelled *peptide E or K* (in 100 mM glucose solution) at room temperature. The GUVs were observed by confocal laser scanning microscopy (Leica TCS SP5, Germany). DOPE-ATTO 633 was excited at 633 nm and detected between 641–700 nm and Oregon Green 488 was excited at 488 nm and detected between 497–574 nm. Any cross-talk was eliminated by using a sequential excitation and emission cycles of both dyes.

## Supplementary information

Supplementary Information
