# Supplementary material for: Interaction of SNARE Mimetic Peptides with Lipid bilayers: Effects of Secondary Structure, Bilayer Composition and Lipid Anchoring
Source: Sci Rep. 2019 May 22;9:7708. doi: 10.1038/s41598-019-43418-w (PMC6531448; doi:10.1038/s41598-019-43418-w)
Supplement: Supplementary file 1 — Supplementary Information [file 41598_2019_43418_MOESM1_ESM.pdf]

Supporting Information Interaction of SNARE Mimetic Peptides with Lipid bilayers: Effects of Secondary Structure, Bilayer Composition and Lipid Anchoring

Swapnil Wagle, Vasil Georgiev, Tom Robinson, Rumiana Dimova, Reinhard Lipowsky and Andrea Grafmüller *

Unbiassed simulations of peptide K and peptide E with the PC bilayer

To understand how the peptides are affected by their interactions with the lipid bilayer, we performed simulations of each of the two peptides in the vicinity of lipid bilayers with two different compositions corresponding to compositions popularly used in two different fusion assays: a neural PC bilayer, which is rich in DOPC, and a negatively charged PG bilayer containing DOPG. The helical peptides were simulated with a random initial orientation close to the bilayers.

In two out of the three simulations, ***peptide K*** remains in solution throughout the simulation, forming only transient contacts with the bilayer. In the third case*, peptide K* absorbs only weakly on the bilayer with the cysteine residue at its C-terminal, with the side chain protruding into the lipid tail region, as shown in the simulation snapshot in Figure S1A. Although the random coil residues – GGGGC- fluctuate in and out of the bilayer, the cysteine does not leave the membrane long enough for the peptide to reorient, so that in this case, the additional residues act similar to a membrane anchor. The rest of the peptide remains solvent exposed despite this cysteine 'anchor'. The orientation of the helix remains flexible with respect to the membrane, although orientations normal to the bilayer, i.e. forming an angle between 0^0 and 45^0 with the bilayer normal, are slightly more favorable than those parallel to the bilayers, and make up ≃60% of the simulation time.

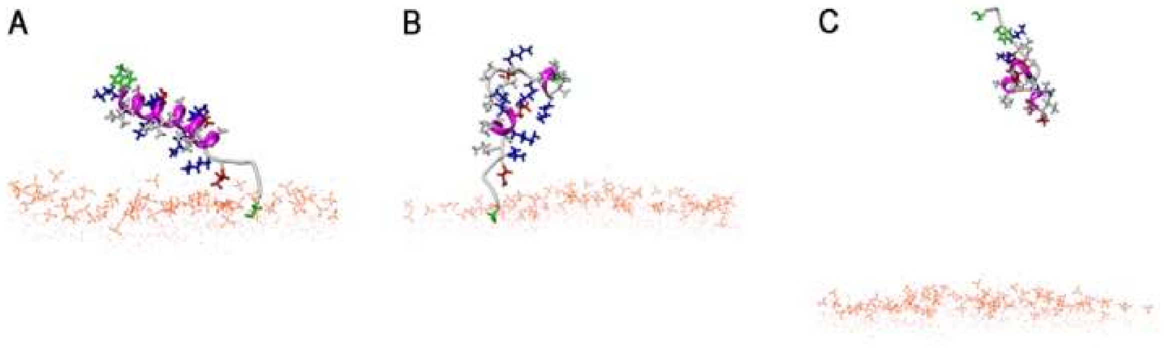

Figure S1: Simulation snapshots of unbiased simulations of the peptides with the PC bilayer (A) and (B) The C-terminal cysteine of *Peptide K* interacting with the PC bilayer residue: (A) initial insertion and (B) unfolded *peptide K* at the end of 1µs simulation; (C) *Peptide E* close to the bilayer.

In all simulations, the peptide partially unfolds, with the largest unfolding observed in the simulation where the peptide C-terminal adsorbs to the bilayer. Here, the helicity of the peptide is reduces to 76% in the 600 ns run, starting from the heptad repeat near the C-terminal. However, because the helicity does not reach a constant value, the simulation time was extended to one microsecond. During the additional 400 ns, unfolding continues until only individual turns of the helix remain, as shown in the snapshot in Figure S1B. The unfolded peptide still remains predominantly in solution. Only the C-terminal GLU and LYS residues interact with the bilayer and stay at average distances of 2.7±0.2 nm and 3.1±0.2 nm from the bilayer midplane, respectively. The GLU side chain forms a hydrogen bond

with the NH_3^+ head group of a DOPE lipid, while the C-terminal lysine side chain can form two hydrogen bonds with PO_4^- moieties of both DOPE and DOPC.

Peptide E does not interact with the bilayer surface in any of the three simulations, and quickly moves to the solvent region. The peptide helicity decreases with simulation time, and adopts a random coil structure (Figure S1C), similar to the behavior observed in the simulations in solution.

Unbiased simulations of peptide K and peptide E with the PG bilayer

The peptide bilayer interactions observed for this composition are shown to be significantly enhanced. In the unrestrained simulations, both peptides adsorb at the bilayer water interface.

Peptide K typically absorbs quickly starting from its C-terminal end as for the PC bilayer. The C-terminal GLU residue interacts with the membranes early on, followed by parallel alignment with the bilayer. The GLU side chains interact with the Ca^{2+} ions in the head-group region acting as a bridge to the PO_4^- groups. In addition, hydrogen bonds form between the LYS side chains and the–OH groups in DOPG head groups as well as with the PO_4^- and less frequently with the choline group and –OH of sphingosine. In contact with the PG bilayer, the peptide maintains its helicity to a larger extent. The extended peptide sequence and the N-terminal tryptophan again interact most strongly with the bilayer. The final orientation on the membrane and the insertion depth (2.8-3.4 nm) vary between independent simulation runs and are determined by the adsorption process rather than representing equilibrium structures. The most shallow insertion depth of 3.4±0.3 nm is observed for the case, where the hydrophobic side chains face the bilayer.

The peptide has aligned with the bilayer after about 190 ns and 120 ns, for the first two simulations when all three GLU residues attain similar distances of 3-3.5 nm from the bilayer midplane. In the last of the three simulations, the peptide adsorbs with the hydrophobic chains facing the bilayer after ~150 ns. The C terminal GLU residue embeds in the head-group region, whereas the other charged residues remain at a larger distance of 3.5-4.0 nm from the bilayer midplane, reflecting weaker peptide absorption and a more flexible orientation. The peptide partially unfolds, because there are fewer stabilizing interactions and hydrogen bonds.

The net-negatively charged ***peptide E*** also adsorbs to the bilayer surface, starting from the C-terminal end. In two of the simulations, the peptide comes rapidly into contact with the bilayer after 6 ns and 84 ns, respectively, and aligns parallel to the bilayer plane at the head group-water interface after 125 ns and 170 ns in the two cases. The charged amino acid side chains of the peptide interact with the bilayer head groups and the Ca^{2+} ions located in the head-group area, preventing the hydrophobic side chains from fully inserting in the lipid tail region. The adsorption of the peptide is strengthened by hydrogen bond formation between GLU and LYS side chains and the –OH in the DOPG head groups and the PO_4^- moieties of DOPG and PSM. In the third simulation the peptide diffuses in the solution phase for about 530 ns before it adsorbs to the bilayer. While in solution, it loses its helicity almost completely. Upon contact with the membrane, the aromatic side chains insert in the bilayer, while the rest of the bulky unstructured peptide remains in solution, and forms much fewer contacts with the bilayer than the helical peptides (Figure 6F).

Adsorption to a larger bilayer patch

To investigate the possibility of finite size effects simulations of the peptides adsorbing to a PG bilayer patch with twice as much area were performed. Again, both peptides adsorb to the bilayer surface. *Peptide K* spontaneously embeds into the bilayer starting from the N terminus and aligns at the head-group tail interface with the hydrophobic chains facing the bilayer forming an angle ~87^0 with the

bilayer normal. The conformation, shown in the Figure S2, is very similar to the conformation predicted for *peptide K* in a PC bilayer, including also the lysine 'snorkle' effect. The hydrophobic side chains insert slightly deeper into the bilayer, reaching a distance 2.3 nm from the bilayer midpane, compared to the deepest insertion of z=2.5 nm observed for the smaller patch. The negatively charged glutamic acid side chains remain at the bilayer-water interface. Similarly, adsorption of *peptide E* also starts from the N-terminal with the hydrophobic side chains oriented towards the bilayer and the deepest insertion (2.7 nm) of the peptide is observed.

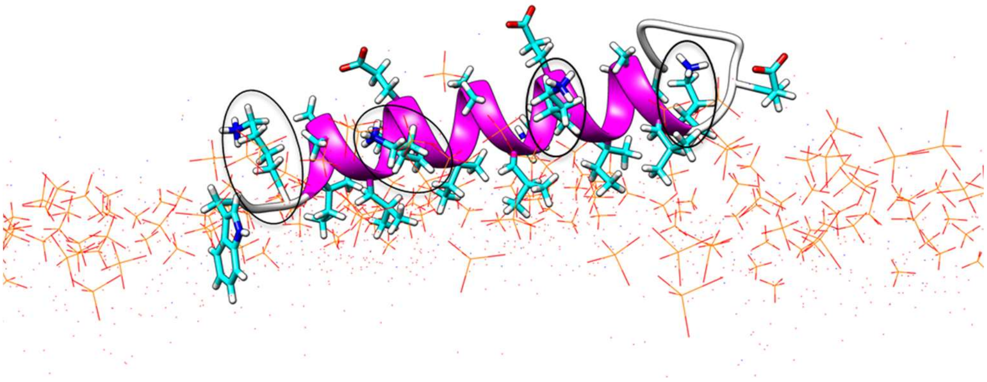

Figure S2: *Peptide K* adsorbed to a larger PG bilayer patch. The peptide backbone is shown in magenta (helical part) and light grey (random coil). Side chains are drawn in stick representation, with the snorkeling lysine side chains marked by black circles.

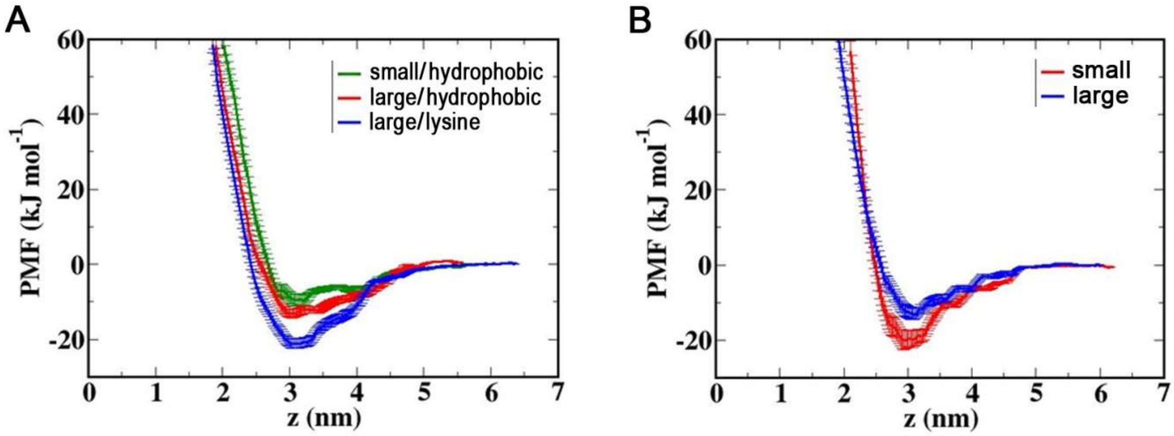

Figure S3: PMF profiles comparing different bilayer sizes and orientation for (A) *Peptide K* . The different colored profiles correspond to the PMFs calculated for: green: a small bilayer patch containing 258 lipids with the peptide facing the bilayer with the hydrophobic face; red: a larger bilayer patch containing 512 lipids with the peptide facing the bilayer with the hydrophobic face, and blue: the larger bilayer patch with the peptide facing the bilayer with negatively charged lysine chains. and (B) *Peptide E.* The colors correspond to: red: the small bilayer patch, blue: the larger bilayer, patch. Both profiles were calculated for the peptide facing the bilayer with the hydrophobic face.

PMF Histograms

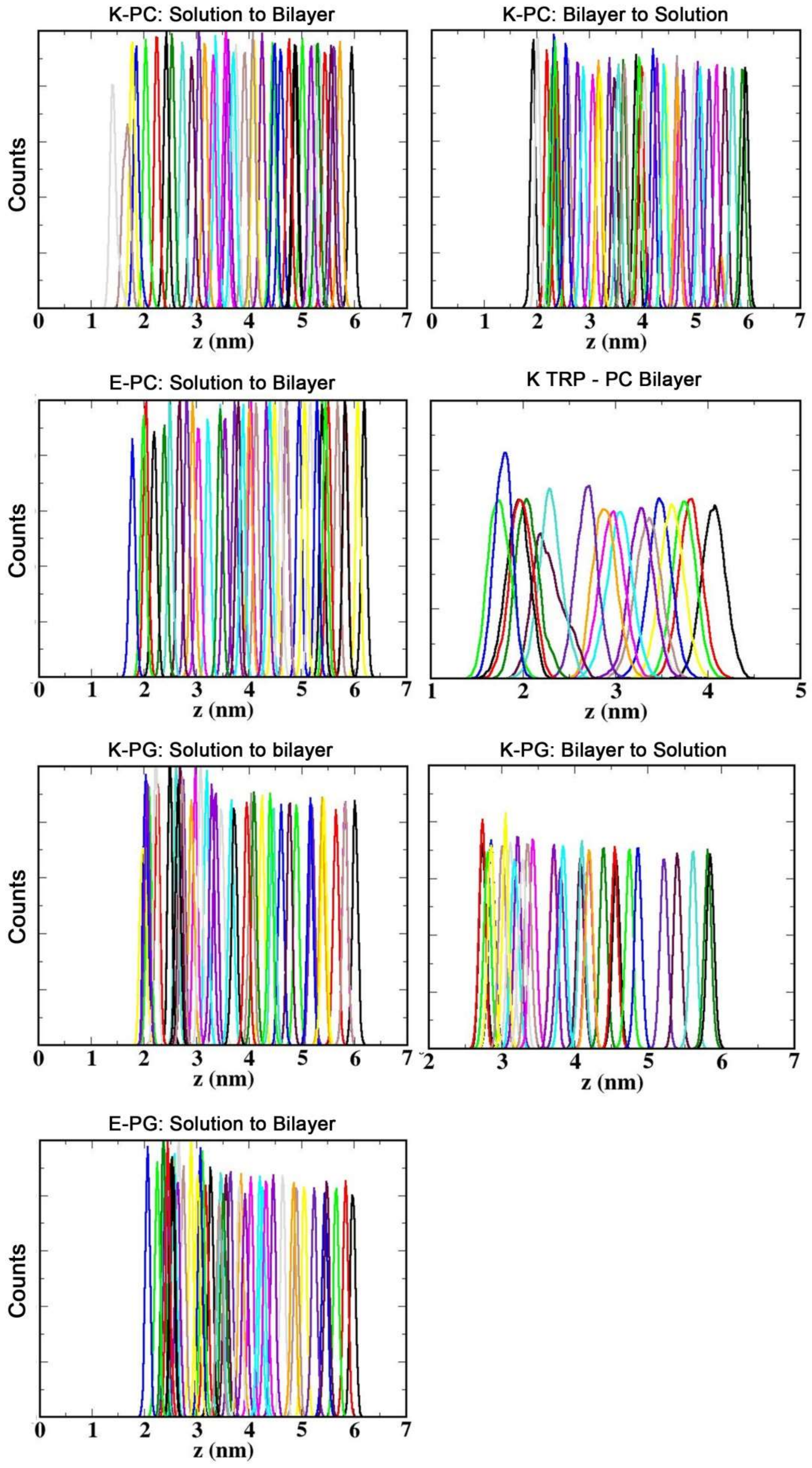

Figure S3: The histograms corresponding to the different PMF in the manuscript.
